# Tetranectin: Molecular Mechanisms, Biological Functions and Clinical Implications

**DOI:** 10.3390/ijms27156732

**Published:** 2026-07-28

**Authors:** Sarfraz Ahmed, Sana Iram, Amar Akash, Kwonyoung Kim, Jihoe Kim

**Affiliations:** Department of Medical Biotechnology, Yeungnam University, Gyeongsan 38541, Republic of Korea; sarfrazahmed6954@yu.ac.kr (S.A.); sanairam157@gmail.com (S.I.); amarakash@yu.ac.kr (A.A.); cathykimva@naver.com (K.K.)

**Keywords:** tetranectin, CLEC3B, plasminogen, extracellular matrix, proteolysis, biomarker, cancer

## Abstract

Tetranectin (TN), also known as C-type lectin domain family 3 member B (CLEC3B), is a secreted glycoprotein originally identified through its high-affinity interaction with plasminogen. Although initially linked to fibrinolysis, TN is now increasingly recognized as an extracellular regulator associated with matrix remodeling, bone development, tissue repair, and disease-related proteolytic processes. Dysregulated TN expression has been reported in a broad range of pathological conditions, including cancer, cardiovascular disease, inflammatory disorders, and neurodegenerative states. In this review, we summarize current knowledge of TN with emphasis on its molecular architecture, ligand-binding properties, trimeric organization, and regulatory mechanisms. We further discuss its context-dependent biological functions, with particular attention to how tissue-specific and compartment-specific TN patterns may influence disease progression and clinical interpretation, especially in oncology. In addition, we evaluate the evidence supporting TN as a diagnostic and prognostic biomarker and consider its emerging therapeutic relevance. Finally, we outline the major challenges that currently limit clinical translation and highlight key directions for future investigation.

## 1. Introduction

The extracellular matrix (ECM) is no longer regarded as a passive structural scaffold, but rather as a dynamic and instructive microenvironment that regulates cell behavior, tissue architecture, and organ homeostasis [1,2,3,4]. A central feature of ECM function is the spatial and temporal control of extracellular proteolysis, which permits physiological tissue turnover, regeneration, and morphogenesis while limiting excessive degradation, fibrosis, or invasive remodeling [5,6]. Proteins that organize protease activity within defined extracellular niches are therefore important determinants of both normal tissue maintenance and disease progression [7,8].

Tetranectin (TN), also designated C-type lectin domain family 3 member B (CLEC3B), has emerged as one such extracellular regulator [9]. TN was originally purified from human plasma and identified through its high-affinity binding to the kringle 4 domain of plasminogen [10]. Subsequent studies expanded its biological relevance beyond fibrinolysis by linking TN to osteogenesis [11], wound repair [12], myogenesis [13], and extracellular remodeling [14]. These observations suggest that TN should not be viewed merely as a plasminogen-binding protein, but rather as a multifunctional extracellular factor that may help organize local proteolytic events.

Structurally, TN occupies a distinctive position within the C-type lectin superfamily. Although it contains a C-type lectin-like domain (CTLD), TN differs from classical carbo-hydrate-recognizing lectins in that it primarily binds protein ligands in a calcium-dependent but carbohydrate-independent manner [15,16,17]. In addition, TN forms a stable homotrimer, a feature that likely underlies its ability to cluster ligands and concentrate proteolytic activity within specific extracellular microenvironments [9,15]. This multivalent organization provides a plausible mechanistic basis for its proposed role as a local scaffold for matrix-associated proteolysis.

Clinical interest in TN has increased because altered TN expression has been report-ed across diverse disease settings. In cancer, both reduced and increased TN levels have been associated with disease progression, depending on tumor type, tissue compartment, and stromal context [10,18,19,20,21,22,23]. Beyond oncology, TN has been implicated in fracture healing, cardiovascular remodeling, inflammatory disease, and neurodegenerative phenotypes [14,24,25,26,27,28]. However, despite growing interest, several important questions remain unresolved, including how TN is regulated across tissues, which binding partners define its disease-specific functions, and why its biological significance appears to differ so markedly among pathological contexts.

This review provides a critical synthesis of current knowledge on TN biology. We discuss its molecular structure and regulation, examine its roles in extracellular proteolysis and tissue remodeling, and evaluate its translational relevance as a biomarker and potential therapeutic target. Particular attention is given to the context-dependent nature of TN, which is likely central to both its biological complexity and its clinical potential.

## 2. Molecular Characteristics of TN

TN is a homotrimeric plasminogen-binding protein present in both blood plasma and the extracellular matrix [9]. Its overall organization is consistent with a protein specialized for extracellular ligand recruitment and localized proteolytic regulation [17]. Structural analyses indicate that TN contains an N-terminal region involved in oligomerization and heparin binding, together with a C-terminal carbohydrate recognition domain/C-type lectin-like domain (CRD/CTLD) responsible for ligand recognition [15,16].

### 2.1. Genomic Organization and Primary Structure

**Figure 1:**
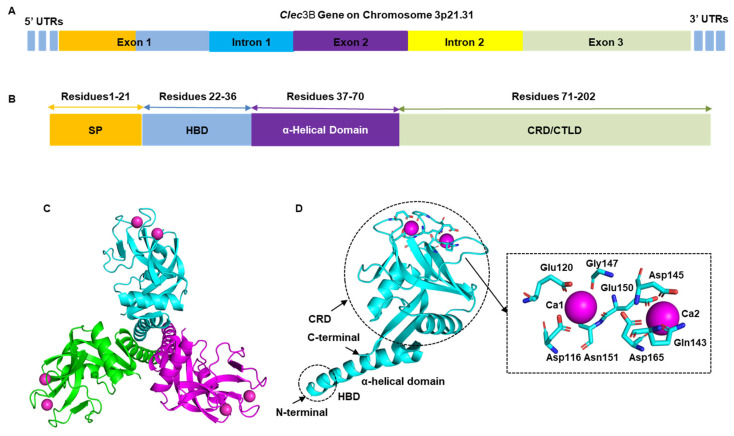
Genomic organization and structure of tetranectin. (**A**,**B**) Schematic representation of the human CLEC3B gene on chromosome 3p21.31, and the corresponding domain structure of TN. The gene contains three exons, two introns, and untranslated regions (UTRs), and encodes a precursor protein with an N-terminal signal peptide (SP), a short heparin-binding domain (HBD), an α-helical region, and a carbohydrate recognition domain/C-type lectin-like domain (CRD/CTLD). (**C**,**D**) Homotrimeric (each monomer in green, cyan and magenta) and momomeric structures of TN, respectively. The box in (**D**) is for the residues involved in calcium coordination within the CRD. Structural visualization was generated using PyMOL (version 3.1.4.1), based on the TN crystal structure (PDB ID: 1HTN). Domain annotation was referenced from NCBI structural resources.

The human CLEC3B gene is located on chromosome 3p21.31, a region frequently implicated in genomic instability and deletions in several malignancies [10,29,30]. The gene spans approximately 12 kb and contains three exons, two introns, and untranslated regions at both the 5′ and 3′ ends (Figure 1A) [31]. It encodes a 202-amino acid precursor polypeptide that enters the secretory pathway through an N-terminal signal peptide (Figure 1B). Following signal peptide cleavage, the mature protein consists of structurally and functionally distinct regions. The N-terminal segment contains a short lysine-rich heparin-binding region and an α-helical domain important for oligomerization. The C-terminal CRD/CTLD mediates binding to the kringle 4 domain of plasminogen in a calcium-dependent manner [16,17,30,31]. Although TN belongs to the C-type lectin superfamily, it appears to use a non-canonical ligand-binding mechanism, as its best-characterized interactions involve protein ligands, rather than classical carbohydrate recognition [32]. This functional divergence highlights the versatility of the CTLD scaffold in extracellular proteolytic regulation.

### 2.2. Trimeric Assembly and Post-Translational Heterogeneity

A hallmark of TN biology is its stable homotrimeric assembly under physiological conditions. Trimerization is primarily mediated by the N-terminal coiled coil region and is likely critical for multivalent ligand recruitment (Figure 1C). The transition from a monomeric to a trimeric state is therefore not merely structural; it is likely central to the functional behavior of TN in extracellular remodeling contexts. Each CRD coordinates calcium ions that contribute to domain stability and ligand-binding competence. The Ca^2+^ in the CRD, present at calcium binding site 1 (Ca1) and calcium binding site 2 (Ca2) and their respective interacting residues are Glu120, Gly147, Asp116, and Asn151, and site 2 Ca^2+^ interacts through Asp145, Gln143, and Asp165, while Glu150 shows common interactions with both the sites (Figure 1D) [16]. The trimeric scaffold of TN firmly arranges three carbohydrate recognition domains (CRDs) around a central triple α-helical coiled coil, creating a defined three-dimensional cluster, rather than three independent binding sites. TN exhibits sequential and structural similarity with C-type lectin family [9,15].

Beyond trimerization, post-translational modification may add further complexity to TN biology. In particular, glycosylation is likely to influence TN folding, extracellular stability, and susceptibility to degradation [33,34,35]. Disease-associated variation in glycosylation may also modify TN localization or ligand interactions, although the functional significance of specific TN glycoforms remains insufficiently characterized and requires direct experimental investigation [36].

### 2.3. The TN Interactome: From Plasminogen to the ECM Scaffold

Plasminogen remains the best-characterized TN ligand, particularly through interaction with its kringle 4 domain [10,17,37]. By recruiting plasminogen into proximity with tissue-type plasminogen activator and urokinase-type plasminogen activator, TN may facilitate localized plasmin generation at sites of active tissue remodeling [38,39]. This provides a mechanistic framework for understanding how TN may influence matrix turnover, fibrinolysis, wound repair, and invasive remodeling.

Importantly, TN is unlikely to act exclusively through the plasminogen axis. Additional reported TN-associated molecules include fibrin, angiostatin, heparin-like ligands, hepatocyte growth factor (HGF), and other extracellular binding partners [40,41,42]. In addition, small molecules such as epigallocatechin gallate have been investigated as modulators of TN-associated functions [43]. Taken together, these observations support the view that TN may act as a multifunctional extracellular scaffold whose activity depends not only on abundance, but also on localization, oligomeric state, post-translational status, and the partner landscape present in a given tissue microenvironment.

## 3. Spatiotemporal Regulation of TN Expression

**Figure 2:**
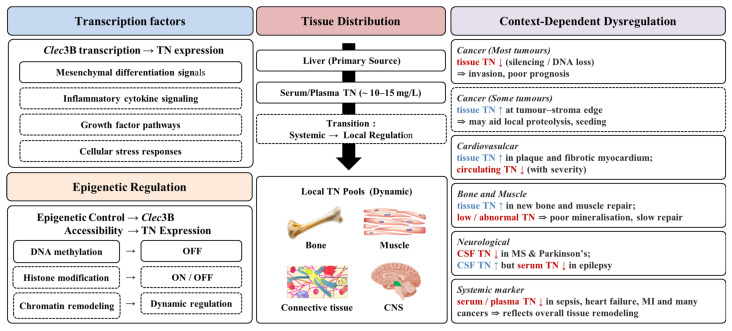
Multilevel regulation of tetranectin expression across tissues and diseases. The left panel outlines regulatory inputs, including differentiation-associated, inflammatory, and stress-related cues, together with transcription factors and epigenetic mechanisms. The middle panel illustrates the relationship between the circulating TN pool and locally regulated tissue pools. The right panel highlights context-dependent dysregulation in cancer, cardiovascular disease, bone and muscle repair, and neurological or inflammatory conditions. Solid boxes indicate experimentally well-supported relationships, whereas dashed boxes indicate proposed or insufficiently established relationships. TN level, ↓ Decrease; and ↑ Increase.

TN expression is regulated across developmental, physiological, and pathological settings, consistent with its proposed role in tissue remodeling, rather than constitutive housekeeping function. Because TN exists in both circulating and tissue-associated pools, its biology is shaped by regulatory events occurring at multiple levels, including transcriptional control, epigenetic modulation, tissue-specific synthesis, and disease-dependent redistribution (Figure 2).

### 3.1. Transcriptional and Epigenetic Landscapes

The transcriptional regulation of CLEC3B appears to be strongly context-dependent (Figure 2, upper-left panel). TN expression has been reported to increase in association with mesenchymal differentiation, osteogenic programs, and matrix remodeling states [11,44]. Available studies further suggest that CLEC3B expression is responsive to growth factor and inflammation-associated cues, although the direct promoter architecture underlying these responses remains incompletely defined [30,45,46,47].

Epigenetic mechanisms may provide an additional explanation for the marked variability in TN expression observed across diseases (Figure 2, lower-left panel) [10,48]. Promoter-associated methylation and related epigenetic alterations have been implicated in reduced CLEC3B expression in several malignancy-associated contexts, particularly those involving abnormalities at 3p21.31 [45,49]. Histone modification and chromatin remodeling may also contribute to context-dependent variation in TN expression, although direct mechanistic evidence in TN biology remains limited [10]. Overall, these findings suggest that TN expression is dynamically reprogrammed according to tissue state and pathological environment.

### 3.2. Physiological Tissue Distribution and Systemic Homeostasis

Under physiological conditions, TN shows broad but heterogeneous tissue distribu-tion (Figure 2, middle panel) [10,50]. Available evidence suggests that the liver is a major source of circulating TN, which is detected at relatively stable concentrations in human serum and plasma [51,52,53]. At the same time, local TN synthesis has been reported in tissues characterized by active remodeling, particularly bone, skeletal muscle, and connective tissues [13,54,55].

TN has also been detected in the central nervous system and cerebrospinal fluid, suggesting a possible role in extracellular proteolytic processes relevant to neural matrix remodeling [56,57,58]. The coexistence of circulating and locally regulated TN pools may be an important feature of TN biology and may help explain its compartment-dependent behavior in disease [24,59].

### 3.3. Dysregulation in Pathological Environments

The context-dependent nature of TN becomes especially evident under pathological conditions (Figure 2, right panel) [10]. In oncology, TN expression often follows a compartment-dependent pattern. In many solid tumors, intratumoral TN is reduced, potentially through genomic loss or epigenetic silencing, and this reduction has frequently been associated with aggressive disease features and poor outcome [23,45,49,60]. By contrast, elevated TN expression or deposition in stromal or invasive compartments has been linked in some settings to enhanced matrix remodeling and metastatic behavior [41,48,60]. These findings are not necessarily contradictory; rather, they likely reflect differences in tissue compartment, cellular source, and disease stage.

TN dysregulation is not restricted to cancer. Altered TN expression or circulating lev-els have also been reported in cardiovascular disease, bone and tendon repair disorders, inflammatory states, and neurological conditions [11,24,58,61]. Because TN is secreted, tissue-level changes may be reflected in serum, plasma, synovial fluid, or cerebrospinal fluid, but the interpretation of such measurements is unlikely to be uniform across compartments [28,53,56,57]. This complexity is central to the translational challenge of TN biology.

## 4. Pleiotropic Biological Functions of TN

**Figure 3:**
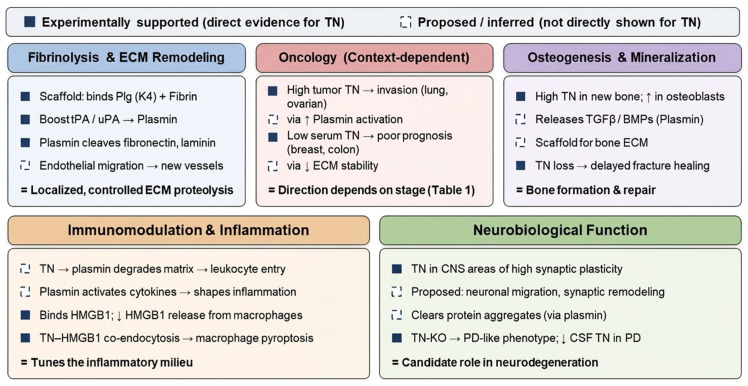
Multifaceted biological functions of tetranectin. This schematic summarizes the major functional domains currently associated with TN, including fibrinolysis/ECM remodeling, osteogenesis and mineralization, inflammation/immunomodulation, oncology, and neurobiological processes. Solid elements represent functions or interactions supported more directly by experimental evidence for TN, whereas dashed elements denote proposed or inferred mechanisms, including steps extrapolated from plasmin-related biology. The figure emphasizes the context-dependent nature of TN function and distinguishes better-established extracellular interactions from more speculative downstream consequences. Abbreviations: ECM, extracellular matrix; tPA, tissue-type plasminogen activator; uPA, urokinase-type plasminogen activator; HMGB1, high-mobility group box 1; BMP, bone morphogenetic protein; CSF, cerebrospinal fluid. ↓ Decrease; ↑ Increase.

The diverse biological activities attributed to TN can be conceptually unified by its ability to organize localized extracellular proteolysis and matrix interactions [10,62,63]. Rather than acting as a classical signaling ligand, TN appears to influence tissue behavior by modulating the spatial coupling of proteases, substrates, matrix components, and regulatory factors [48,63]. The major biological functions currently attributed to TN, together with the distinction between experimentally supported and proposed mechanisms, are summarized in Figure 3 [11,13,62]. This framework helps explain its reported involvement in fibrinolysis, skeletal remodeling, inflammation, cancer progression, and neural tissue dynamics.

### 4.1. Fibrinolysis and ECM Remodeling

The best-established role of TN lies within the plasminogen activation system. TN binds plasminogen and appears to facilitate its local activation to plasmin, thereby supporting localized proteolysis at the cell surface and within the extracellular matrix [64]. This mechanism supports the view that TN functions as a regulator of matrix turnover, rather than as a structural ECM component.

By recruiting plasminogen into proximity with fibrin and plasminogen activators such as tPA and uPA, TN may enhance protease-rich remodeling within restricted extracellular sites [40,42]. Localized plasmin generation contributes to degradation of structural ECM proteins, including fibronectin and laminin, and supports controlled matrix turnover during wound healing, angiogenesis, and tissue regeneration [65,66,67]. Disruption of this proteolytic balance may contribute to pathological outcomes, including fibrosis, defective repair, or excessive tissue degradation.

### 4.2. Osteogenesis and Mineralization

TN is enriched in mineralized tissues and has been implicated in bone development and skeletal repair [11]. Its expression increases during osteoblast differentiation and fracture healing, supporting the view that TN is linked to active osteogenic remodeling [11,68].

Experimental evidence suggests that TN may promote matrix mineralization through multiple mechanisms [11]. By influencing plasmin-mediated remodeling, TN may facilitate the release or activation of matrix-bound growth factors, such as transforming growth factor-β (TGF-β) and bone morphogenetic proteins (BMPs), thereby supporting osteogenic signaling [54,68,69]. TN may also contribute to the organization of the bone extracellular environment in ways that favor mineral deposition. Consistent with these observations, TN deficiency has been associated with delayed fracture healing and impaired skeletal repair [54].

### 4.3. Immunomodulation and the Inflammatory Response

Although TN is not a classical immune receptor, it may influence inflammatory tissue dynamics by modulating extracellular protease activity and matrix accessibility [70]. Plasmin-mediated degradation of the interstitial matrix facilitates immune cell infiltration into inflamed tissues, and TN may contribute indirectly to this process through local regulation of plasminogen activation.

TN may also influence selected aspects of extracellular inflammatory signaling through plasmin-associated proteolysis. In addition, recent work has connected TN to the HMGB1 axis in sepsis, where lower TN levels have been reported in patients, and TN-targeted interventions affect survival in experimental models [27,71,72,73]. These findings suggest that TN may participate in inflammation-associated extracellular regulation, although its precise molecular role remains incompletely defined and likely varies by disease context.

### 4.4. Context-Dependent Roles in Oncology

The role of TN in cancer is best understood as compartment-dependent, rather than uniformly tumor-suppressive or tumor-promoting (Table 1) [41,60]. In one recurring pattern, TN expressed by tumor cells themselves is reduced through epigenetic silencing, genomic loss, or altered exosomal packaging [23,48,49]. In some experimental systems, reduced CLEC3B or TN expression levels have been associated with altered AMPK–VEGF signaling, epithelial–mesenchymal transition-related changes, or p38/ERK pathway activity; however, these mechanisms appear to be tumor-type-specific, rather than universal [74]. Clinically, lower intratumoral or circulating TN has been linked in several cancers to advanced disease or poor prognosis [20,22,23,29,45,49,75].

A local gain of TN within stromal or invasive niches is often associated with cancer-associated fibroblasts [46]. In such settings, plasminogen-associated and matrix-remodeling activities that normally support tissue repair may instead contribute to extracellular matrix breakdown, pro-angiogenic remodeling, and tumor cell migration [46,60,64,77]. Thus, the biological significance of TN in cancer cannot be inferred from expression level alone. Rather, it must be interpreted in relation to tissue compartment, cellular source, disease stage, and the prevailing proteolytic state of the tumor microenvironment [81,82,83]. The microenvironmental factors that may influence TN-associated biology in tumors are summarized in Table 2.

Circulating TN is generally reduced in several cancers and has been proposed as a non-invasive biomarker, but its direction of change may diverge from tissue-associated TN [18,19,21,89]. Reduced circulating TN may reflect local sequestration, altered extracellular turnover, or disturbed proteolytic balance during progressive tumor remodeling [81,83]. Collectively, these findings underscore that the clinical interpretation of TN in oncology must be compartment-specific and tumor-type-specific.

### 4.5. Neurobiological and Systemic Perspectives

Evidence for TN function in the central nervous system remains more limited than in bone or cancer, but several observations are suggestive. TN has been detected in cerebrospinal fluid and in neural regions associated with high synaptic plasticity, implying a possible role in extracellular proteolytic processes relevant to neural remodeling [25,26,56,58,90]. TN may participate in plasmin-related extracellular events within neural tissues; however, its direct contribution to neuronal migration, synaptic plasticity, or aggregate clearance remains to be clarified. TN has also been investigated in Parkinson’s disease-related contexts [25,26,58]. Experimental studies in TN-deficient models and biofluid analyses suggest that TN may be relevant to neurodegenerative phenotypes, although the mechanistic basis remains incompletely resolved [10,26,56,57].

More broadly, TN has been associated with cardiovascular and metabolic disease settings [24,30]. In heart failure, coronary artery disease, acute myocardial infarction, and dialysis-associated stress, altered circulating TN levels have been reported, while tissue-associated TN may increase in fibrotic remodeling environments [24,91,92]. These observations support the idea that TN may serve as a remodeling-sensitive biomarker, but larger and better-controlled prospective studies will be required to establish its clinical value.

## 5. Clinical Implications of TN

At present, the clinical relevance of TN appears to be better supported in the biomarker setting than in direct therapeutic application [61]. Because TN is measurable in multiple biofluids and is linked to extracellular remodeling, fibrosis, and proteolytic balance, it offers translational appeal across oncology, inflammation, cardiovascular disease, and tissue repair [24,61,92,93]. However, this same breadth creates interpretive complexity.

### 5.1. Tetranectin as a Diagnostic and Prognostic Biomarker

TN possesses several analytical features that support its evaluation as a biomarker candidate, including extracellular accessibility, relative stability, and measurable presence in serum and plasma. Nevertheless, these advantages must be weighed against the clinical literature, which remains heterogeneous in design and inconsistent in reported associations.

A recurring observation in oncology is that low circulating TN is associated with poor prognosis. In metastatic breast cancer and other tumor types, reduced serum or plasma TN has been linked to advanced disease, impaired treatment response, or shorter survival [21,79,80,89]. One interpretation is that reduced circulating TN reflects local sequestration or altered extracellular turnover within diseased tissues, although this remains hypothetical [10,81,83]. Importantly, this pattern cannot be generalized to tissue-associated TN, which may increase in certain tumor compartments and may therefore carry different biological implications.

Outside oncology, altered TN levels have also been reported in rheumatoid arthritis, cardiovascular disease, diabetes, and other remodeling-associated conditions. These findings suggest that TN may be most useful as a remodeling-sensitive biomarker within multimarker frameworks, rather than as a disease-specific standalone diagnostic test [24,94,95,96]. A major challenge, however, is that the biological meaning of TN differs across serum, plasma, synovial fluid, cerebrospinal fluid, and tissue compartments [28,56,58]. Clinical interpretation will therefore require strict compartment-specific validation.

### 5.2. Therapeutic Potential: Targeting the TN–Plasminogen Axis

The extracellular location of TN makes it an attractive theoretical therapeutic target, particularly in diseases characterized by dysregulated proteolysis or matrix remodeling. In cancer, selective disruption of TN-mediated plasminogen recruitment could, in principle, attenuate invasive remodeling and angiogenesis [63,97]. In inflammatory disease, especially sepsis, targeting the TN–HMGB1 axis has shown encouraging preclinical signals [27,72]. In addition, exogenous TN or TN restoration strategies have been explored in experimental neurodegenerative and repair-related settings [70,98].

However, broad systemic manipulation of TN is unlikely to be straightforward. Because TN participates in multiple physiological processes, including bone homeostasis, wound healing, and extracellular repair programs, indiscriminate inhibition could be detrimental [12,28]. Any future therapeutic strategy will therefore likely require selective, disease-context-specific modulation of TN-associated pathways, rather than nonspecific TN blockade.

### 5.3. Challenges and Barriers to Clinical Translation

Several practical barriers currently limit the clinical development of TN. First, substantial inter-study variability complicates comparison across cohorts and disease settings. Differences in sample type, pre-analytical handling, assay design, patient selection, and analytical sensitivity all influence reported TN levels. Standardization of laboratory methods will therefore be essential before TN can be meaningfully benchmarked across studies [99].

Second, the context-dependent biology of TN complicates threshold-based interpretation. Both increased and decreased TN may be clinically relevant, depending on disease stage, tissue compartment, and underlying remodeling state. Third, most available data remain cross-sectional and associative. Larger longitudinal studies, ideally anchored to mechanistic biology and clear compartment-specific stratification, will be needed to determine whether TN provides independent value beyond existing biomarkers, or whether its best use lies in integrated multimodal models [61].

## 6. Future Perspectives and Research Directions

A central challenge in TN research is to move from descriptive association toward mechanistic resolution. Progress will depend on distinguishing when TN functions primarily as a scaffold, when it serves as a circulating readout of remodeling, and when it directly shapes disease biology. Addressing this question will require better structural, spatial, and compartment-specific approaches.

### 6.1. Incomplete Structural Definition of Ligand-Binding

Although the TN trimer and isolated CTLD have been structurally characterized, no high-resolution structure of full-length TN in complex with major ligands, such as plasminogen, tPA, or relevant ECM partners, has been established. As a result, the geometry and conformational logic of these interactions remain incompletely defined. High-resolution TN–ligand structures obtained by co-crystallography or cryo-electron microscopy would substantially improve mechanistic interpretation and may enable more rational therapeutic targeting.

### 6.2. Unresolved Physiological Relevance of Carbohydrate Recognition

Whether TN retains a physiologically meaningful carbohydrate-binding function remains uncertain. Although TN belongs to the C-type lectin superfamily, available evidence more strongly supports protein–ligand recognition than canonical glycan binding [100,101]. Systematic glycan array screening and co-structural studies will be required to determine whether carbohydrate recognition contributes to signaling, cellular interactions, or disease-specific functions under defined conditions [102].

### 6.3. Deciphering the Extended TN Interactome

The TN field remains heavily centered on plasminogen, yet existing data suggest a broader and incompletely mapped interactome. TN has been reported to associate with extracellular regulatory proteins such as tPA and HGF, but the full range of tissue-specific TN-associated complexes remains unknown [40]. Affinity purification–mass spectrometry, glycoproteomics, structural biology, and spatial proteomics should therefore be prioritized to define context-resolved TN interaction networks.

Equally important is the need to understand how TN-associated extracellular events connect to intracellular responses. Whether TN-associated proteolysis is coupled to protease-activated receptors, integrin-linked pathways, or other ECM-sensing mechanisms remains largely unresolved and represents a major mechanistic gap in the field.

### 6.4. Spatial Biology and Cell-Type Specificity

The paradoxical roles attributed to TN in cancer and inflammatory disease likely arise, at least in part, from insufficient spatial resolution. It remains unclear in many settings whether the dominant TN source is tumor cells, stromal fibroblasts, hepatocyte-derived circulation, immune cells, or reparative tissue populations. This distinction is critical because the meaning of TN expression depends heavily on its cellular origin and extracellular destination.

Single-cell transcriptomics, spatial transcriptomics, and compartment-resolved proteomics may help clarify these issues. In parallel, conditional knockout and knock-in animal models will be needed to dissect tissue-specific TN functions in bone, muscle, nervous tissue, and tumor-associated stroma. Such approaches are likely to be more informative than further bulk expression studies alone.

### 6.5. Translational Roadmap and Standardization

Future translational studies should prioritize interpretability over simple detectability. Measuring TN is not sufficient; its clinical significance must be anchored to tissue source, biological compartment, disease stage, and remodeling state. Standardized reference ranges will need to be matrix-specific, and biomarker studies should be designed prospectively, with clearly defined stratification variables [103].

TN is unlikely to become a universal standalone biomarker. Its greater value may lie in multimodal integration with imaging, tissue pathology, genomics, and complementary remodeling markers. This will be particularly relevant in diseases where extracellular matrix dynamics strongly influence progression, therapeutic resistance, or tissue repair outcomes.

## 7. Conclusions

TN is increasingly recognized as a multifunctional regulator of extracellular proteolysis and matrix remodeling, rather than merely a plasminogen-binding protein. Its trimeric structure, non-canonical ligand-binding properties, and broad disease associations support a model in which TN functions as a context-sensitive extracellular scaffold whose biological significance varies by tissue compartment and pathological state.

At the same time, the field remains limited by incomplete interactome definition, compartmental ambiguity, and insufficient mechanistic resolution. The key challenge is no longer simply to document where TN is altered, but to determine how, where, and under what conditions those alterations become functionally meaningful. With improved structural insight, spatially resolved biology, and analytical standardization, TN may emerge as a useful biomarker of pathological remodeling and, in selected contexts, a therapeutically relevant extracellular target.

## Figures and Tables

**Table 1:** Compartment-dependent patterns of tetranectin expression and their reported clinical relevance in human cancers.

Cancer Type	TN Source & Expression	Proposed Mechanism	Evidence Level	Clinical Significance	Key References
Group A: Tumor cell TN loss (Tumor-suppressor mode)					
Hepatocellular carcinoma	Tumor cells/exosomes Downregulated	Downregulation of TN inhibits AMPK and upregulated VEGF; promotes EMT and angiogenesis	level 2 + 3 (in vitro functional; IHC/serum cohort; no in vivo tumor model)	Low TN indicates poor survival	[29,74]
Lung adenocarcinoma	Tumor cells Downregulated	Downregulation allows EMT; altered immune infiltration (largely inferred)	Level 3 + 4 (IHC; in silico)	Low TN indicates poor survival	[23]
Clear cell renal cell carcinoma	Tumor cells, copy number loss Downregulated	Antiproliferative; Inhibits p38/ERK MAPK	Level 2 + 3 (in vitro functional; IHC)	Low TN indicates poor survival	[49]
Breast	Tumor/serum Downregulated	Reflects tumor burden; inhibits EMT signaling pathway	Level 3 (observational)	Low TN indicates advanced stage; poor survival	[21,76]
Pancreatic	Tumor/Serum Downregulated	Reduced serum TN; Estrogen-induced matrix remodeling and antitumor conditioning	Level 3 + 4 (serum proteomic; multi-omics)	Low serum TN linked to pancreatic cancer	[22,45]
Oral squamous cell carcinoma	Tumor, serum, saliva Downregulated	Sequester plasminogen, limiting ECM breakdown (proposed)	Level 3 (observational)	Low TN linked to metastasis and poor survival	[20,75]
Group B: Stromal/CAF-derived TN gained (Tumor-promoter mode)					
Colorectal	CAF-secreted (stroma) Upregulated	Drives tumor cell migration and actin remodeling	Level 2 + 3 (knockdown + rescue in vitro; IHC)	High stromal TN with α-SMA indicates poor survival	[46]
Gastric (intratumoral)	Downregulated overall Upregulated intratumorally	Plasmin-driven ECM breakdown and angiogenesis	Level 3 (IHC; single cohort; direction disputed)	High intratumoral TN indicates poor survival	[60,77]
Bladder	Stromal (in situ), present/high	Not established; stromal TN proposed to aid invasion	Level 3 (IHC, stromal)	High stromal TN predicts shorter recurrence-free survival	[78]
Group C: Circulating TN (Biomarker-dominant)					
Ovarian	Serum Downregulated	Not established; compartments diverge within tumor	Level 3 (serum + stromal IHC cohorts, *n* up to ~445)	Low serum TN indicates poor prognosis	[19,79]
Colorectal (plasma)	Plasma Downregulated	Not established	Level 3 (plasma)	Low plasma TN indicates advanced disease	[18,80]

Abbreviations: CAF, cancer-associated fibroblast; EMT, epithelial–mesenchymal transition; IHC, immunohistochemistry; TN, tetranectin. Evidence level: Level 1, in vivo tumor model evidence; Level 2, functional in vitro evidence; Level 3, human observational evidence; Level 4, in silico or bioinformatic evidence only. Where two levels are indicated, both evidence types are available. Note: Reported associations vary by tumor type, tissue compartment, and study design; therefore, the biological interpretation of TN should not be generalized across cancers without context-specific validation.

**Table 2:** Tumor microenvironmental variables that may influence tetranectin-associated biology.

Tumor Microenvironment Variables	Likely Influence on TN Biology	Key Supporting Concept	Key References
Protease activity (plasmin, MMPs)	Increases TN processing and local plasminogen activation, favoring ECM remodeling and invasion.	Higher proteolysis intensifies TN-dependent pericellular proteolysis.	[10,64]
Stromal density and fibrosis	Promotes accumulation and retention of TN in the tumor stroma, limiting its circulating levels.	Dense stroma sequesters TN and concentrates its matrix-anchoring role.	[60,84]
Hypoxia and angiogenic signaling	May enhance invasive and proteolytic programs, shifting TN toward a pro-invasive context.	Hypoxia-driven invasion often coincides with intensified matrix remodeling.	[10,84]
ECM composition (glycosaminoglycans, heparin-like motifs)	Stabilizes TN localization and strengthens its interaction with plasminogen and matrix components.	Glycosaminoglycan-rich ECM increases local TN concentration and functional avidity.	[37,42,85]
Immune and myeloid infiltration	Alters local cytokine and protease release, indirectly modulating TN-mediated matrix turnover and fibrinolytic activity.	Inflammatory cells reshape the proteolytic and matrix landscape around the tumor.	[30,86]
Tumor stage and invasive front geometry	Mature, invasive fronts show higher stromal and matrix TN, whereas early or encapsulated lesions exhibit lower deposition.	TN distribution tracks the spatial organization and aggressiveness of the tumor.	[87,88]

Abbreviations: ECM, extracellular matrix; MMPs, matrix metalloproteinases; TN, tetranectin. Note: The variables listed here are intended as conceptual modifiers of TN-associated behavior in tumors. In several cases, the supporting rationale derives from the broader ECM or proteolysis literature, rather than from TN-specific mechanistic evidence alone.

## Data Availability

No new data were created or analyzed in this study. Data sharing is not applicable to this article.
